# Early synaptic dysfunction of striatal parvalbumin interneurons in a mouse model of Parkinson’s disease

**DOI:** 10.1016/j.isci.2024.111253

**Published:** 2024-10-24

**Authors:** Quansheng He, Xiaowen Zhang, Hongyu Yang, Dahui Wang, Yousheng Shu, Xuan Wang

**Affiliations:** 1 Institute for Translational Brain Research, State Key Laboratory of Medical Neurobiology, MOE Frontiers Center for Brain Science, MOE Innovative Center for New Drug Development of Immune Inflammatory Diseases, Fudan University, Shanghai 200032, China; 2 School of Systems Science, Beijing Normal University, Beijing 100875, China; 3 Department of Neurology, Affiliated Hospital of Jiangsu University, Zhenjiang, Jiangsu 212001, China

**Keywords:** Cellular neuroscience, Neuroscience, Pathophysiology

## Abstract

In Parkinson’s disease (PD), the loss of dopaminergic signaling remodels striatal circuits, causing abnormal network activity. The timing and impact on various striatal cell types during this reorganization are unclear. Here we demonstrate that dopamine depletion rapidly reduces parvalbumin (PV) expression. At the synaptic input level, PV interneurons shift toward inhibition in the excitation-inhibition balance early on, a week before a similar shift in spiny projection neurons (SPNs). At the cellular level, both PV interneurons and SPNs experience a significant decrease in their spiking and bursting rates, respectively, which corresponds to a reduction in gamma and beta (*early beta*) oscillations during the early stage of PD. Importantly, the pharmacogenetic activation of PV interneurons reverses gamma deficits and suppresses beta (*late beta*) oscillation in the striatum of parkinsonian mice. Collectively, our findings underscore the vulnerability of PV interneurons to dopamine depletion and their responsibility for the evolution of abnormal activities in parkinsonian striatum.

## Highlights

• Dopamine depletion rapidly reduces parvalbumin expression in the striatum
• Dopamine depletion induces an earlier synaptic E-I balance shift in PV-INs compared to SPNs
• PV-INs spike less as network oscillations change early in dopamine depletion
• Activating PV-INs reverses gamma deficits and suppresses beta oscillation in PD mice

## Graphical abstract

**Figure:**
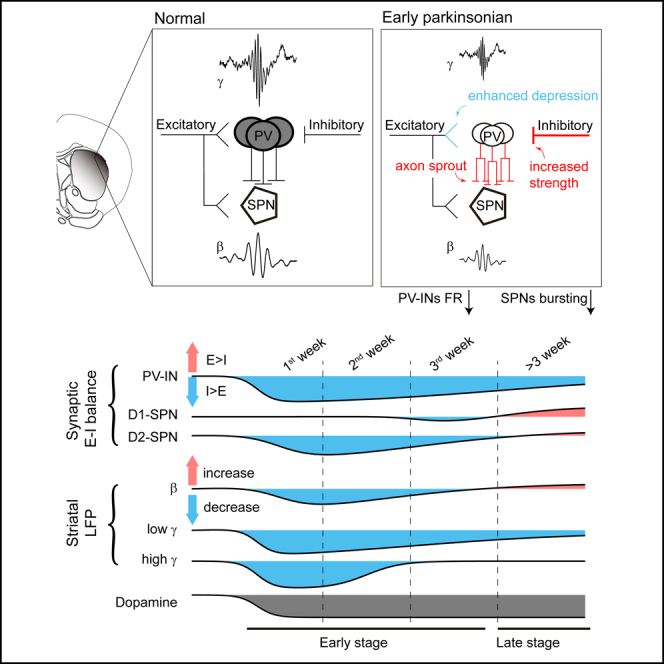

## Introduction

Parkinson’s disease (PD) is characterized by the progressive degeneration of midbrain dopaminergic neurons, a process that is intimately linked to the emergence of motor deficits.^1^ The loss of dopamine has a ripple effect throughout the brain, with the striatum being one of the most notably affected regions.^2^^,^^3^ Proper motor behavior relies on the coordinated activity of striatal circuits, which are densely innervated by midbrain dopaminergic neurons and exhibit rich network activity including beta and gamma oscillations.^4^ Following dopamine depletion in PD, striatal circuits undergo profound reorganization in both cellular and synaptic excitability, leading to abnormal network activity in the striatum.^5^^,^^6^ Despite the significant strides made in understanding these changes at the micro-level, the overarching connection between these microscopic alterations and the macroscopic network behavior of the striatum in the context of PD remains a complex puzzle.

The dopamine receptors D1 and D2 are selectively expressed in two types of spiny projection neurons (SPNs) in the striatum. Consequently, these SPNs respond in opposite ways to dopamine depletion: D1-expressing SPNs (D1-SPNs) experience a reduction in synaptic drive, while D2-expressing SPNs (D2-SPNs) show an increase.^7^^,^^8^ The divergence results in an imbalance in the output of striatal and basal ganglia circuits^9^^,^^10^^,^^11^ (but see^12^^,^^13^^,^^14^ for different views).

Interestingly, changes in synaptic inputs to D1-and D2-SPNs are not temporally aligned in PD, despite a synchronized loss of dopamine signaling. For example, D2-SPNs show a decrease in spinal density early after dopamine depletion, whereas D1-SPNs show this decrease after two months.^15^ This temporal discrepancy raises intriguing questions: Why do the onset times of these changes differ? Do they change independently within the circuit? Could other cell types, which may undergo changes prior to the SPNs, be orchestrating the circuit’s reorganization? The striatum also contains various types of interneurons, whose contribution to PD pathology cannot be ignored.^16^ It is therefore essential to identify the primary cell type that is both susceptible to dopamine depletion and plays a pivotal role in modulating synaptic plasticity and network dynamics within the striatum. Despite the importance of understanding these processes, a comprehensive time course analysis of the synaptic changes in PD is currently lacking in the literature.

Parvalbumin-expressing interneurons (PV-INs) are the predominant subtype of GABAergic interneurons in the striatum. They receive input from cortical and thalamic sources, controlling the activity and plasticity of SPNs via powerful feedforward mechanisms.^7^^,^^17^^,^^18^ This process generates striatal gamma oscillation and suppresses beta rhythms generated by SPNs.^19^^,^^20^^,^^21^ Additionally, these interneurons have been linked to various neurological disorders.^22^^,^^23^ However, their involvement in PD progression remains underappreciated.

To explore the progression of striatal circuit plasticity following dopamine loss, we studied synaptic and network activity alterations in a mouse model of PD by injecting 6-hydroxydopamine (6-OHDA) into the medial forebrain bundle (MFB). Our data show that 6-OHDA treatment rapidly modifies the cytological features of PV-INs, including a decrease in PV expression. Within this early time window, electrophysiological studies *in vitro* and *in vivo* reveal a shift to increased inhibition in PV-INs, accompanied by a decrease in their firing rate and striatal gamma and beta (*early beta*) oscillations. Pharmacogenetic activation of PV-INs reverses gamma deficits and suppresses beta (*late beta*) oscillation in the parkinsonian striatum, suggesting that the activation of PV-INs may provide potential therapeutic benefit by normalizing the dynamics within the striatal network. Unexpectedly, our time-resolved recordings reveal a recovery trend from surgical stress in sham-operated mice, a trend that is absent in parkinsonian mice. Collectively, these results underscore the susceptibility and influence of PV-INs on striatal circuitry in PD, suggesting their potential role in promoting circuit resilience, which may represent an early target in the pathogenesis of PD.

## Results

### Dopamine depletion causes a rapid reduction in parvalbumin expression in the striatum

**Figure 1:**
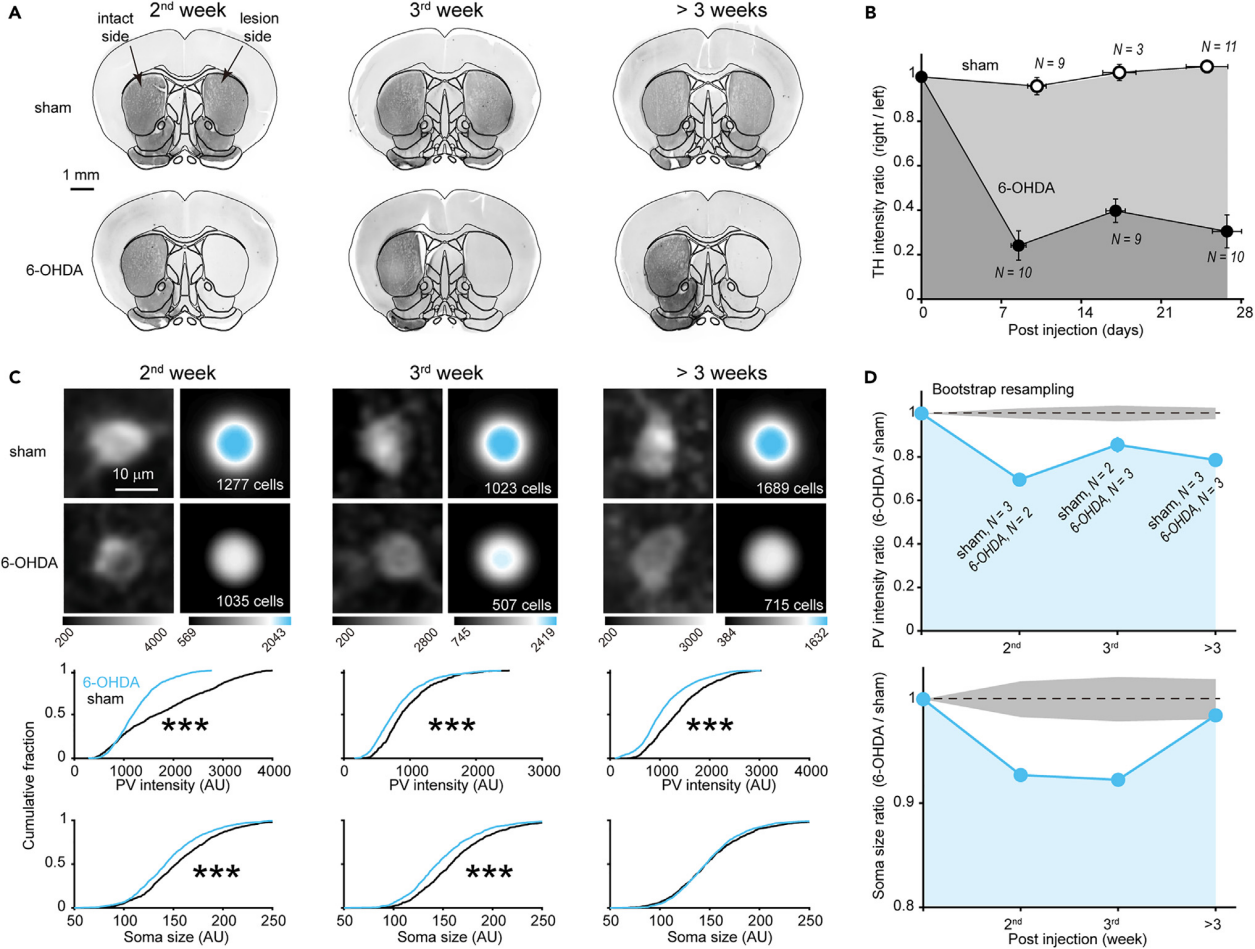
Dopamine depletion induces a rapid reduction in PV expression in the striatum (A and B) TH immunostaining shows the time course of dopaminergic fiber denervation following the injection of 6-OHDA or vehicle into the right MFB. Scale bar, 1 mm. The number of animals is shown in the figure. Error bars indicate s.e.m. (C) Changes in PV expression and PV-INs soma size in the 6-OHDA group. Images: PV immunostaining image of a single PV-positive soma (left column) or average across somata (right column). Below: cumulative distribution of PV intensity and soma size (∗∗∗*p* < 0.001, Kolmogorov-Smirnov test, the number of cells is shown in the figure). Scale bar, 10 μm. (D) Time course of changes in PV expression and soma size. Shading gray area indicates ±3 SD from the mean of bootstrap samples. In panels B and D, the initial point on each curve, marked at a y axis value of 1, serves as an artificial reference point introduced to aid in visualizing the effects of 6-OHDA. See also Figure S1.

One early characteristic of PD is the loss of dopaminergic axon terminals in the striatum.^24^ To determine the time course of dopaminergic denervation in the striatum, we injected 6-OHDA or vehicle into the right MFB to generate parkinsonian or sham-operated animals, respectively. At various time points after injection, we performed immunostaining of the striatum for tyrosine hydroxylase (TH), a selective marker of dopaminergic neurons. Consistent with previous studies in mice,^25^^,^^26^ the infusion of 6-OHDA into the MFB resulted in a rapid loss of ipsilateral striatal TH immunoreactivity within the first week (Figures 1A and 1B).

To investigate the response of PV-INs to dopamine depletion, we performed immunostaining for PV, which was found to be associated with the synaptic excitability of PV-INs.^27^ We found that the intensity of PV was significantly reduced in 6-OHDA-treated mice (Figure 1C). Similar to TH intensity, the time course plot showed a rapid decrease in PV intensity during the 2^nd^ week and then remained at low levels (Figure 1D), suggesting a close dependence of PV expression on dopamine level. In addition, we found that the soma size of PV-INs significantly reduced during the early time windows (the 2^nd^ and 3^rd^ week) and then gradually recovered >3 weeks after injection (Figures 1C and 1D). The density of PV-INs was consistent across all examined time windows (Figure S1). Considering axons of PV-INs have been shown to sprout during the 1^st^ week after dopamine depletion,^28^ the reduction in soma size may represent a parallel phenomenon, confirming the vulnerability of PV-INs morphology to dopamine depletion.

### Distinct remodeling trajectory of excitatory-inhibitory (E-I) balance in parvalbumin-expressing interneurons

The reduction in PV protein level suggests the presence of early synaptic dysfunction in PV-INs.^27^^,^^29^ We next performed *in vitro* whole-cell recordings to examine the synaptic alterations in PV-INs as well as in SPNs, with the help of transgenic mice (D1-CreAi9, D2-CreAi9 and PV-CreAi9), or AAV viruses (AAV2/9-Ef1a-DIO-EYFP) injected into Cre mice. We recorded neurons in the dorsolateral striatum (Figure S2), a subregion known for its abundance of PV-INs and its involvement in sensorimotor functions.^30^ To minimize possible dopamine-depletion independent effects at the very early stage (<1 week),^31^ we focused our study during the 2^nd^, 3^rd,^ and >3 weeks (weeks 4–8) after injection. We refer to the 2^nd^ and 3^rd^ week as *early* time windows.

**Figure 2:**
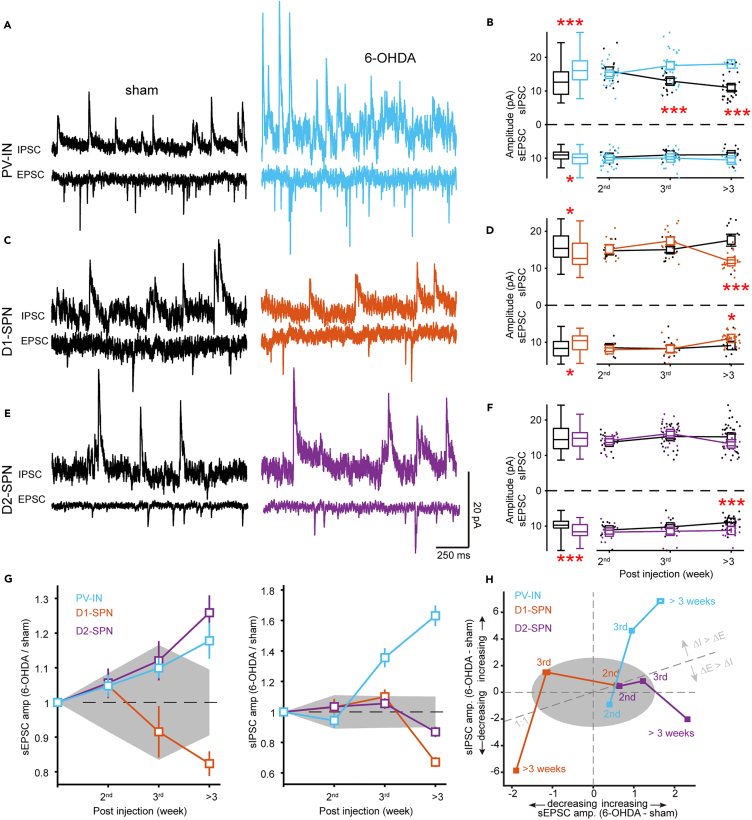
Distinct remodeling trajectory of E-I balance in PV-INs (A) Exemplary traces of sEPSCs or sIPSCs from PV-INs in sham-operated mice or mice >3 weeks after 6-OHDA injection. Note that the traces in panels (A), (C), and (E) are scaled identically. (B) Pooled data across all time windows (box graphs) or pooled by specific time window (line graphs). Notice the early increase in sIPSC amplitude of PV-INs. (∗*p* < 0.05, ∗∗*p* < 0.01, ∗∗∗*p* < 0.001, Welch’s *t*-test). Error bars indicate s.e.m. (C–F) Similar to (A-B), displaying data from D1-SPNs and D2-SPN, respectively. Error bars indicate s.e.m. (G) Relative changes in sEPSC (left) and sIPSC (right) amplitudes. Error bars indicate ±3 SD (noise level) from bootstrap samples. Shading area represents the maximum noise level among cell types. The initial point on each curve, marked at a y axis value of 1, serves as an artificial reference point. (H) Time course of the relative amplitude changes for sEPSC (x axis) and sIPSC (y axis), demonstrating the evolution of E-I balance. See also Figure S2.

We recorded spontaneous excitatory postsynaptic currents (sEPSCs) and spontaneous inhibitory post-synaptic currents (sIPSCs), with the membrane potential camped at −70 mV and 0 mV, respectively. Figure 2 summarizes the results of peak amplitudes (for additional parameters such as frequency and waveform kinetics, refer to Figure S2). For PV-INs, when considering all the data across time windows, we observed a significant increase in the amplitude of both sEPSCs and sIPSCs in the 6-OHDA group (Figures 2A and 2B). However, a closer examination revealed that the sIPSCs amplitude progressively increased from the 3^rd^ week onward. While the sEPSC amplitude also displayed an increasing trend, it did not achieve statistical significance within any of the investigated time windows. One might notice the temporal changes in the sham group, potentially reflecting the sham-operated animals' inherent capacity to recuperate from surgical procedures (see later in discussion and see discussion). It should be noted that during the 2^nd^ week, when PV deficits became apparent, neither sEPSC nor sIPSC amplitudes changed in PV-INs, suggesting the contribution of other synaptic properties associated with the deficits within this time window (see later in discussion).

It is of interest to know the changes in the amplitude of both sEPSCs and sIPSCs in SPNs. Similar to PV-INs, differences in SPNs between sham and 6-OHDA groups manifest within specific time windows, and pooling data from all windows may obscure such differences. For D1-SPNs, pooled analysis revealed that both sEPSC and sIPSC amplitudes were significantly reduced in the 6-OHDA group, while further analysis found that this reduction specifically occurred >3 weeks after injection but not in other time windows (Figures 2C and 2D). These results again emphasize the importance of examining of specific time window. For D2-SPNs, there was an increase in sEPSC amplitude in the 6-OHDA group, a change that became significant only >3 weeks after injection (Figures 2E and 2F).

To make the changes in amplitude more visually explicit, we calculated the relative changes, i.e., by normalizing the data in the 6-OHDA group to that of the sham group. A value of 1 means no change, greater than 1 indicates an increase, less than 1 indicates a decrease (Figure 2G). The sEPSC amplitude of D1-SPNs continued to decrease following the lesion, whereas that in D2-SPNs persisted in increasing. These patterns align with the modulatory influence of dopamine on synaptic inputs at D1 and D2 receptors.^32^ To our surprise, in PV-INs that express D1-type receptors,^33^ sEPSC amplitude progressively increased over time, similar to D2-but not D1-SPNs (Figure 2G left). One possible explanation for this counterintuitive result is that inhibitory synapses actually dominate the adaptation process, such that the E-I balance mechanisms overwhelm the influence of D1-type receptors on the amplitude of sEPSC. In line with the explanation, we found that sIPSC amplitude increased earlier and to a greater extent than that of sEPSC (Figure 2G right).

To illustrate the relative shifts in the excitatory-inhibitory (E-I) balance, we calculated the differences between the E and I amplitudes of the 6-OHDA group and those of the sham group (Figure 2H). In this two-dimensional diagram, we observed that different cell types exhibited distinct temporal trajectories of E-I balance changes spanning into different portions of the diagram. From the 2^nd^ to 3^rd^ week, the two types of SPNs showed opposite changes in E, with D1-SPNs decreasing while D2-SPNs increasing. From the 3^rd^ week onward, both types of SPNs showed a decrease in I. In contrast, PV-INs followed a distinct trajectory. They initially experienced an increase in I, and this was followed by a subsequent rise in E.

In summary, our data demonstrate that dopamine depletion induces systematic changes in both excitatory and inhibitory synaptic input strength. To our surprise, SPNs showed delayed changes in E-I balance long after the dopamine depletion, biased toward excitation >3 weeks after injection. Those increased synaptic excitability of SPNs at late time windows may contribute to the exaggerated beta oscillation during locomotion, which we named *late beta*. In contrast, PV-INs showed earlier inhibition-biased changes in synaptic input, suggesting that PV-INs may play a leading role in shaping synaptic adaptations of SPNs via E-I balance mechanisms.

### Enhanced excitatory short-term depression in parvalbumin-expressing interneurons

**Figure 3:**
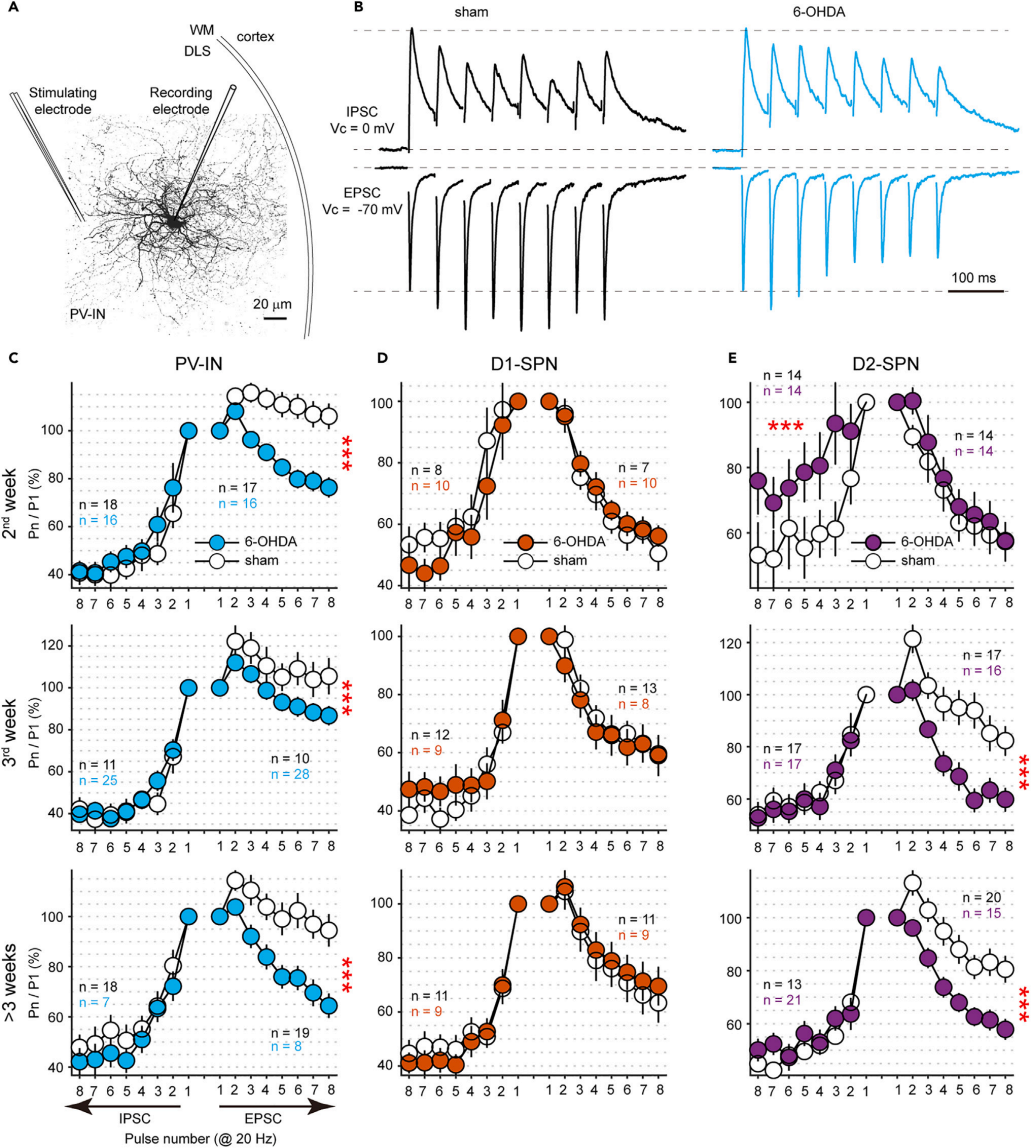
The early enhancement of excitatory short-term depression in PV-INs (A) Schematic of intrastriatal field stimulation. DLS, dorsolateral striatum. WM, whiter matter. (B) Average traces of evoked EPSCs and IPSCs obtained from one PV-IN in sham-operated mice or mice during the 2^nd^ week after injection. These traces have been normalized to their initial peak amplitudes. Notice the enhanced synaptic depression of EPSCs in 6-OHDA-treated mice. (C) Normalized peak amplitudes of EPSCs (rightward) and IPSCs (leftward) evoked by 20 Hz stimulation. (∗∗∗*p* < 0.001, two-way ANOVA. The number of cells in each group is shown in a color-coded manner). Error bars indicate s.e.m. (D and E) Similar as (C) for D1-SPNs and D2-SPNs, respectively. (∗∗∗*p* < 0.001, two-way ANOVA). See also Figure S3.

During the analysis of spontaneous synaptic activity, we found no overt synaptic dysfunction associated with PV deficits during the 2^nd^ week. Next, we studied electrically evoked synaptic activity. We placed the stimulating electrode in the striatum (approximately 100 μm away from the recorded cell) to unbiasedly evoke all possible synaptic inputs to PV-INs (Figure 3A). In PV-INs, the evoked EPSCs of the 6-OHDA group showed a prominent depressive response during the 2^nd^ week, whereas the sham group showed a facilitatory response (Figures 3B and S3A). The short-term plasticity of IPSCs remained unchanged. Such differences were consistently observed across all time windows examined (Figure 3C). Since short-term plasticity, in conjunction with static synaptic strength, dictates the efficacy of synaptic transmission during high-frequency neuronal firing,^34^ we speculate that the dysregulated excitatory presynaptic transmission could be a main cause of abnormal oscillations during the 2^nd^ week (see later in discussion).

Regarding the short-term plasticity of D1-SPNs, no changes were observed across all time windows examined, implying that dopamine depletion does not significantly alter the presynaptic function of synapses onto D1-SPNs (Figures 3D and S3B). Interestingly, D2-SPNs showed a transient reduction in the depression of IPSCs during the 2^nd^ week (Figures 3E and S3C), which may contribute to the decrease in beta power during this early time window (see later in discussion). From 3^rd^ week on, D2-SPNs showed an enhancement of excitatory synaptic depression similar to that of PV-INs, possibly related to the increased amplitude of ESPCs in both these cell types.

Notably, while previous research has also observed differences in the short-term plasticity of D2-SPNs,^35^ our findings suggest that these disparities are more attributable to a recovery effect in the sham group than to an exacerbation of short-term depression in the 6-OHDA group (Figure 3E). This distinction only becomes evident when comparing the data from the 2^nd^ week, underscoring the significance of longitudinal assessments in understanding the dynamics of these neuronal responses.

### Alterations in spiking activity of parvalbumin-expressing interneurons and spiny projection neurons *in vivo*

**Figure 4:**
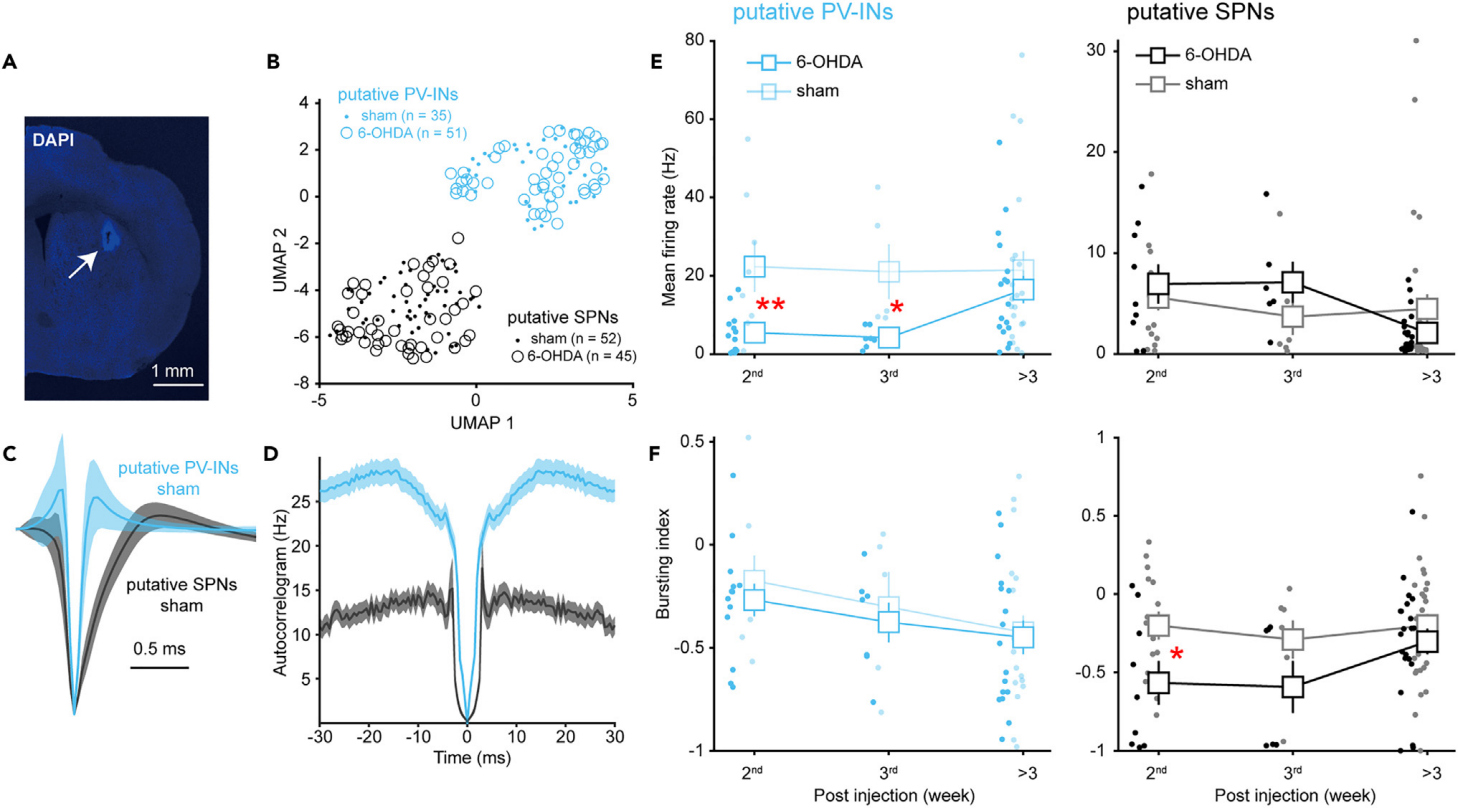
Alterations in spiking activity of striatal neurons *in vivo* (A) Representative coronal slices of the striatum show the recording lesion site (arrow). Scale bar, 1 mm. (B) Separation of striatal neuron subtypes via unsupervised waveform classification (UMAP dimensionality reduction and ISO-SPLIT clustering). Some units recorded in the first week after injection were also included to increase statistical power. Data were recorded from awake, head-fixed mice. (C and D) Average normalized waveforms (C) and autocorrelograms (D) of putative PV-INs and SPNs. Notice that putative PV-INs have narrower spike waveforms and a higher firing rate. (E) Alterations in mean firing rate of putative PV-INs (left) and SPNs (right). (∗*p* < 0.05, Welch’s *t*-test). Error bars indicate s.e.m. (F) Similar as (E) for burst indices. (∗*p* < 0.05, ∗∗*p* < 0.01, Welch’s *t*-test). Error bars indicate s.e.m.

How does the synaptic dysfunction associated with the PV deficit, as described above, affect neuronal spiking output? To address such a question, we performed *in vivo* electrophysiology recording from the striatum of head-fixed awake mice (Figure 4A).

By employing unsupervised classification techniques on spike waveforms of single units, we discerned two neuronal populations (Figure 4B) with distinct waveform duration (Figure 4C) and autocorrelogram profile (Figure 4D). We designated the population characterized by narrower spike waveforms and a higher firing rate as putative PV-INs, while the other as putative SPNs. Time course analysis of the mean firing rate revealed significant declines in the early time windows in PV-INs, a pattern not observed in SPNs (Figure 4E). These decreases in firing rate among PV-INs are fully consistent with the observed increase in their inhibitory synaptic strength and the enhanced excitatory short-term depression. In our examination of bursting activity, we detected a notable reduction specifically in SPNs during the early time window, a phenomenon not seen in PV-INs. While further investigation is warranted, the early reduction in SPNs' bursting activity might be linked to changes in synaptic short-term plasticity. Consistent with previous studies in mice,^36^^,^^37^ we observed no significant alterations in the mean firing rate and bursting for either cell type>3 weeks after injection. Thus, it appears that the dysregulated synaptic input does indeed modify the spiking activity of both PV-INs and SPNs, particularly in the early phases following dopamine depletion.

### Early reduction in beta and gamma oscillations in the striatum following dopamine depletion

**Figure 5:**
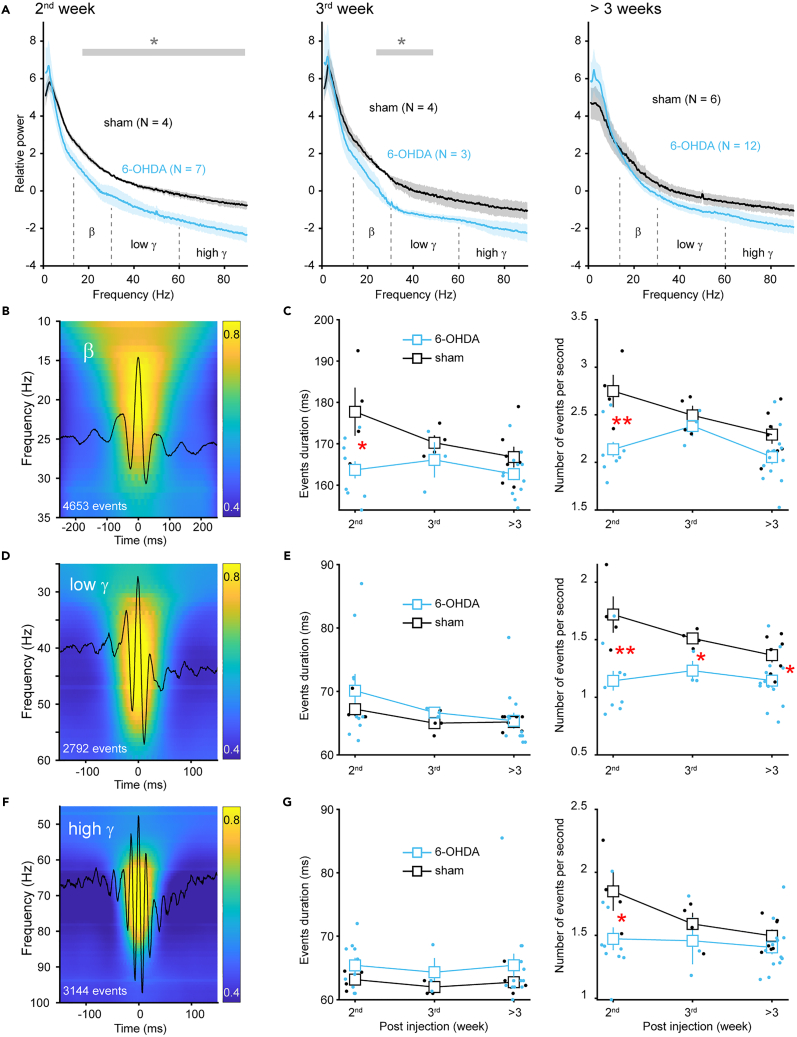
Dopamine depletion induces early beta and gamma deficits in the striatum (A) Power spectrum of striatal LFP at different time windows after 6-OHDA injection. Data were obtained from awake head-fixed mice. The power spectra have been normalized to their respective mean values to account for potential impedance fluctuations that may occur over time. (∗*p* < 0.05, Welch’s *t*-test). (B) Average wavelet spectrogram of raw LFP aligned to the peak of beta burst events. Values are normalized to the maximum value at each frequency before averaging. The overlaid black trace is the average beta band filtered LFP (13–30 Hz). (C) Time course of changes in beta event duration and occurrence rate. Event amplitudes are not shown because of the lack of impedance normalization (∗*p* < 0.05, ∗∗*p* < 0.01, Welch’s *t*-test). Error bars indicate s.e.m. (D and E) Similar to (B-C), showcasing LFP wavelet spectrogram and time course analysis of events for the low gamma (30–60 Hz). Error bars indicate s.e.m. (F and G) Similar to (B-C), but for the high gamma (60–90 Hz). Error bars indicate s.e.m.

Kopell’s team’s pioneering computational models have highlighted the distinct roles of PV-INs and SPNs in the genesis of striatal beta and gamma oscillations,^21^^,^^38^ both of which correlate with symptom severity of PD.^39^ The altered firing patterns could therefore result in abnormal network oscillations during early time windows. Indeed, power spectrum analysis of striatal local field potential (LFP) revealed power deficits in beta (13–30 Hz) and gamma band (30–90 Hz), most prominently observed during the 2^nd^ week and gradually became less apparent >3 weeks after injection (Figure 5A). The specific power of low gamma (30–60 Hz) exhibited a decrease during the 2^nd^ week and continued into the 3^rd^ week (Figure 5A left and middle), aligning with the reduced firing rate of PV-INs within these time windows. Concurrently, the power of high gamma (60–90 Hz) and beta showed transient reductions only during the 2^nd^ week (Figure 5A left), coinciding with the lower firing rate in PV-INs and reduced bursting activity in SPNs. Regarding synaptic input, both cell types displayed an alteration in short-term depression without changes in the amplitude of sEPSCs or sIPSCs during this precise time window (i.e., the 2^nd^ week, as shown in Figures 2 and 3). Therefore, it is speculated that short-term plasticity, rather than static strength, that underpins the synaptic mechanisms linked to the deficits in high-frequency oscillations (beta and gamma) during the very early development of PD.

Further analysis of beta and gamma burst events confirmed those power changes (Figures 5B–5G). Interestingly, event-based analysis additionally showed a significant reduction in low gamma event rate >3 weeks after injection (Figure 5E right), reflecting the different data feature sensitivity between these two analysis methods. Therefore, the changes in network oscillations were consistent with the spiking activity deficits of PV-INs and SPNs, as predicted by computational models.^21^^,^^40^

The reduction in beta oscillation was quite unexpected. Exaggerated cortical-basal ganglia beta oscillation is the established biomarker in human patients with PD and non-human primate PD models.^41^ Previous studies in mice at late time windows (>3 weeks after injection) reported an increase in beta power.^36^^,^^42^ This discrepancy could be due to timing issues, and therefore we named this early reduced beta as *early beta*. Importantly, we noted that the overall reduction in high-frequency oscillations is not due to the reduced motor viability in 6-OHDA-treated mice. Because both sham- and 6-OHDA-treated animals were maintained in a quiet awake state during recording and data segments with motion artifacts were excluded during analysis. Interestingly, similar reductions in beta and gamma power were recently observed in PV knockout mice,^43^ suggesting that the early reductions in beta and gamma oscillations in 6-OHDA-treated mice can be attributable to the PV deficit associated synaptic dysfunction.

### Pharmacogenetic activation of parvalbumin-expressing interneurons increases gamma oscillation and suppresses beta oscillation in parkinsonian mice

Thus far, our data suggests a correlation between PV-IN synaptic dysfunction and abnormal network oscillations. We then used pharmacogenetic tools to interrogate the causal role of PV-INs in the regulation of striatal network oscillations. To this end, we selectively expressed hM3Dq receptors, which can only be activated by the designer drugs (e.g., clozapine N-oxide, CNO), in striatal PV-INs of mice, concurrently with 6-OHDA injection in the MFB. Three weeks post-surgery, we confirmed *in vitro* that CNO elevated the resting membrane potential and firing responses of PV-INs with step current injections (Figure S4). Subsequently, we conducted striatal LFP recordings.

**Figure 6:**
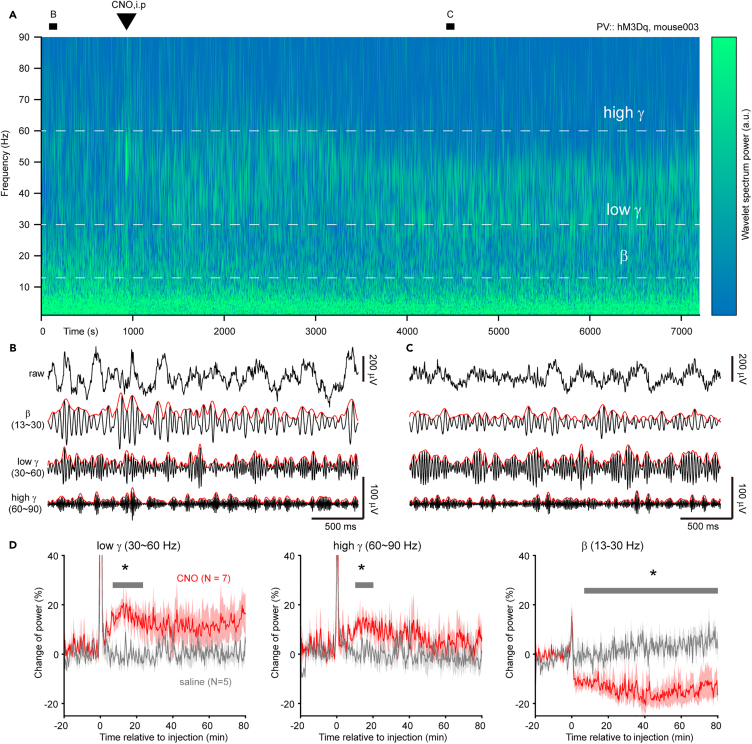
Pharmacogenetic activation of PV-INs reverses gamma deficits as well as suppresses beta oscillation in 6-OHDA-treated mice (A) Wavelet spectrogram of the striatal LFP before and after the intraperitoneal injection of CNO. The mouse received 6-OHDA treatment on the same day that the pharmacogenetic viruses were injected into the striatum, and the LFP was recorded three weeks post-surgery (see STAR Methods). (B and C) 3-s segments of filtered traces before and 1 h after CNO injection, as marked in (A). Envelope in red. (D) Time course of changes in power of different frequency bands following the administration of CNO (red) or saline (gray) in 6-OHDA mice. Shaded area indicate s.e.m. See also Figure S4.

Intraperitoneal injection of CNO (3 mg/kg) rapidly increased the gamma band power within minutes and persisted for at least 1 h (Figures 6A–6D). An hour later, the power of high gamma gradually returned to baseline levels, while low gamma power remained high, indicating that PV-INs participate in the two types of gamma oscillations to different degrees. Interestingly, the CNO application also reduced the power of beta (*late beta*) and the reduction was sustained during the rest of the recording (>2 h). Therefore, we demonstrated the causal role of PV-INs in the regulation of gamma and beta oscillations in the parkinsonian striatum and their potential therapeutic benefits in PD treatment.

## Discussion

Using immunostaining at different time windows after the 6-OHDA lesion, we found a rapid reduction in PV expression. From cell-type specific whole-cell recordings, we found the PV deficit associated with synaptic dysfunction in PV-INs. We also discovered temporal evolution trajectories of synaptic E-I balance in both PV-INs and SPNs, revealing how these changes are tied to their respective alterations in spiking patterns and network oscillations. Utilizing pharmacogenetic tools, we confirmed this connection. Consequently, the synaptic dysfunction associated with the PV deficit emerges as an early hallmark of the 6-OHDA PD model.

As for intrinsic excitability of PV-INs, we observed no change, at least in the 4^th^ week after the lesion, in firing response to the current injection (Figure S5). Of note, we observed that afterdepolarization potentials were more depolarized in PV-INs of 6-OHDA-treated mice, a phenotype similar to PV knockout mice.^44^ This result confirmed once again the reduction in PV expression.

### The vulnerability and capacity of parvalbumin-expressing interneurons in shaping parkinsonian striatal circuits

PV-INs are highly responsive to changes in dopamine levels. At the early stage, we found the PV-IN soma size decreased at the 2^nd^ week, or maybe at a much earlier time that we did not examine. A pioneering study showed that 6-OHDA lesion induced PV-INs axons sprouted within 1 week and those axons formed new synapses specifically onto D2-SPNs.^28^ Consistent with this finding, we observed an increase in sIPSC frequency in D2-but, not D1-SPNs during the 2^nd^ week after the lesion (Figure S2). In addition to the morphology response in PV-INs to dopamine depletion, the affecting aspects include membrane potential,^45^ and gene expression^29^ – both of which have a profound relationship with intracellular Ca^2+^ level that is buffered by PV protein. While comparable sensitivities may exist in other cell types within striatal circuits,^46^ the highest firing rate of PV-INs enables them to transmit signals effectively across the network. Consequently, the early alterations in PV-INs become predominant drivers shaping how other cell types adapt via E-I balance mechanisms. Taking SPNs as an example, the E-I balance mechanisms may counteract the inhibition from PV-INs sprouting axons by strengthening excitatory input, a result that has been observed in D2-SPNs (Figure 2).

The strong inhibition from PV-INs sprouting axons may result in less bursting in SPNs (Figure 4). The sprouting axon of PV-INs has been predicted to increase the bursting of SPNs based on the premise of the unaltered firing pattern of PV-INs.^28^ As opposed to this prediction, one recent study reported the Ca^2+^ activity (probably a result of the bursting of action potentials) of SPNs was decreased in 6-OHDA-treated mice.^42^ Given the unique role of SPNs in the generation of beta oscillations,^38^ we hypothesize that the axon sprouting mechanism contributes to the early reduction in beta oscillation.

How the early dysfunction of PV-INs affect the evolution of synaptic adaptation at the late stage of PD development? At the late stage of PD development in rodent models, researchers found a loss of striatal PV-INs^47^ or a reduction in synaptic innervation onto SPNs from PV-INs.^48^ Overextension and subsequent damage of PV-INs results in excitation-biased synaptic inputs in SPNs at a late stage (Figure 2H). Therefore, we hypothesize that the hypo-functionality of PV-INs at a late stage leads to the disinhibition of SPNs and indirectly contributes to the enhancement of beta oscillation (*late beta*) observed during the locomotion of PD animals. It is worth investigating in the future whether an early pharmacogenetic treatment targeting PV-INs could have a therapeutic effect on slowing the progression of PD.

### On the temporal changes in the sham group

Our time course recordings reveal that the sham-operated animals exhibit a variety of temporal changes in synaptic activity, including 1) decreased in sIPSC amplitude of PV-INs (*p* = 0.006, Kruskal-Wallis test), 2) decreased in sEPSC frequency of PV-INs (*p* = 0.037, Kruskal-Wallis test), 3) decreased in sEPSC amplitudes of D2-SPNs (*p* = 0.045, Kruskal-Wallis test), 4) transition from paired-pulse depression to facilitation in EPSCs of D2-SPNs (*p* = 1.96e-6, Kruskal-Wallis test).

It should be noted that despite these variations, the differences between sham and 6-OHDA groups are consistent with previous research. For instance, Warre et al.,^35^ by recording at 19–30 days (around the 3^rd^ week) after 6-OHDA lesioning, found that D1-, but not D2-SPNs show decreased sEPSC frequency, which corroborates our results. They also reported an increase in short-term depression (STD) of EPSCs in D2-SPNs, a phenomenon mirrored in our data. However, our extended time course recordings suggest that this STD difference is due to a recovery in the sham group’s STD. Furthermore, the prolonged sIPSC decay time in D1-SPNs corroborates the results of Boccalaro et al.^49^ Lastly, the increased sIPSC frequency in D2-SPNs during the 2 nd week aligns with the observations made by Gittis et al.^28^ during the 1^st^ week after 6-OHDA lesion.

How should we interpret the temporal changes observed in sham-operated animals? The short-term plasticity data from D2-SPNs offer intriguing insights. In healthy mice without prior surgery, intrastriatal electrical stimulation induced paired-pulse facilitation of EPSCs in D2-SPNs.^50^^,^^51^ However, in our sham-operated animals, there was a noticeable shift in short-term plasticity: an initial phase of depression in the 2^nd^ week was followed by facilitation in the subsequent weeks. This transition likely reflects the recovery process from surgical intervention in the sham-operated animals. In contrast, this capacity for recovery seems to be impaired in animals treated with 6-OHDA. Surgery, or anesthesia combined with surgery, has been found to have lasting effects on synaptic transmission.^52^^,^^53^ Our findings therefore highlight the significance of longitudinal assessments for comprehending the dynamics of neuronal responses to dopamine depletion. Furthermore, they raise a critical question about how 6-OHDA may disrupt the inherent resilience of striatal circuits.

### Limitations of the study

Firstly, although we demonstrated that striatal PV-INs have an increased inhibitory synaptic input in 6-OHDA-treated mice, we did not identify the main source of this increment. At the circuit level, PV-INs received multiple sources of inhibitory input, including striatal neurogliaform interneurons (NGFs),^54^^,^^55^ thalamic reticular nucleus (TRN),^56^ and globus pallidus externa (GPe).^57^ NGFs are driven by cholinergic interneurons (ChINs), which are overactive in PD.^54^^,^^58^ Therefore, the ChINs**→** NGFs**→**PV-INs pathway, may serve as a good candidate among others. At the receptor level, one possible mechanism could be the disinhibition of presynaptic D2 receptors.^59^ If this were the case, it would be interesting to test whether the IPSC amplitudes would also be increased in ChINs in parkinsonian mice due to the same disinhibitory mechanism.^59^

Secondly, we correlate the synaptic changes of PV-INs and SPNs to the altered beta and gamma band LFP activity in the striatum. However, it is imperative to recognize that this correlation does not represent the sole mechanism at play in generating the abnormal striatal network activity. The contribution from other striatal cell types, as well as neurons in other brain areas, cannot be excluded. Since dopaminergic axons distribute across the basal ganglia–thalamocortical network,^3^ there may be an area showing pathological local activity earlier than, and confounding the present findings in the striatum. For example, the cortex is sensitive to dopamine depletion^60^ and has shown a decrease in mid-gamma oscillation power (41–45 Hz) within hours post-6-OHDA injection, followed by enhanced beta oscillation synchronization between the two hemispheres' cortex over subsequent days.^61^^,^^62^ The rapid and enduring cortical activity changes likely impact striatal function through cortico-striatal synapses, indicating a significant role in striatal network dysregulation. This underscores the need for a comprehensive understanding of the interplay between these brain regions.

Thirdly, the effect of sex on the observed changes has not been examined. Although PD affects both sexes, there are clear sex differences in several aspects. Men, for example, present with a higher incidence and an earlier disease onset than women.^1^ This disparity may be attributed to the neuroprotective advantages that estrogen provides, with studies on 6-OHDA-lesioned animals showing that estrogen offers significant protection, particularly in cases of partial lesions (<60% loss of dopaminergic neurons).^63^ Importantly, estrogen has been shown to upregulate the expression of PV,^64^ hinting at a possible neuroprotective function of PV in the early stage of PD progression. Consequently, further research is imperative to elucidate the sex-specific role of PV in PD pathogenesis.

## Resource availability

### Lead contact

Further information and requests for resources and reagents should be directed to and will be fulfilled by the Lead Contact, Quansheng He (He_quansheng@fudan.edu.cn).

### Materials availability

This study did not generate new unique reagents.

### Data and code availability

• The data of this study are available from the corresponding author upon request.
• The publicly available MATLAB toolboxs used in this study are listed in the key resources table. Additional custom codes are also available from the corresponding authors upon request.
• Any additional information required to reanalyze the data reported in this article is available from the corresponding author upon request.

## Acknowledgments

This study was supported by the National Natural Science Foundation of China Project (NSFC, 32130044 to Y.S., 32100930 to Q.H.), 10.13039/501100002855Ministry of Science and Technology of the People's Republic of China (STl2030-Major Projects 2021ZD0202500 to Y.S.), 10.13039/501100001809NSFC (T2241002 to Y.S.), 10.13039/501100012247Program of Shanghai Academic/Technology Research Leader (21XD1400100 to Y.S.), 10.13039/501100002858China Postdoctoral Science Foundation (2020M681160 to Q.H.).

## Author contributions

X.Z., X.W., and Y.S. initiated the project. X.Z., X.W., and Q.H. performed the experiments. Q.H. performed data analysis. H.Y. and D.W. contributed to the data analysis. Q.H. and X.W. wrote the article.

## Declaration of interests

The authors declare no competing financial interests.

## STAR★Methods

### Key resources table

**Table:** 

REAGENT or RESOURCE	SOURCE	IDENTIFIER
**Antibodies**		

Rabbit anti-TH	Chemicon	Cat# AB152; RRID:AB_390204
Rabbit anti-PV	Millipore	Cat# MAB1572; RRID: AB_2174013
Donkey anti rabbit Alexa Fluo-647	Invitrogen	Cat# A31573; RRID: AB_2536183
Donkey anti rabbit Alexa Fluo-488	Invitrogen	Cat# R37118; RRID: AB_2556546

**Bacterial and virus strains**		

AAV2/9-Ef1a-DIO-EYFP-WPRE-hGH pA	BrainVTA	N/A
AAV2/9-hSyn-DIO-hM3D(Gq)-mCherry-WPRE-pA	Taitool	N/A

**Chemicals, peptides, and recombinant proteins**		

L-Ascorbic acid	Sigma Aldrich	Cat# A4544
6-hydroxy dopamine	Sigma Aldrich	Cat# H4381
desipramine	Sigma Aldrich	Cat# D3900

**Experimental models: Organisms/strains**		

Mouse: PV-IRES-Cre	The Jackson Laboratory	IMSR_JAX:008069
Mouse: Ai9	The Jackson Laboratory	IMSR_JAX:007909
Mouse: D1-Cre	MMRRC	MMRRC_030989
Mouse: D2-Cre	MMRRC	MMRRC_032108

**Software and algorithms**		

SHARCQ	Lauridsen et al., 2022^65^	https://github.com/wildrootlab/SHARCQ
Chronux	Bokil et al. 2010^66^	http://chronux.org/
wlBurst_v2	Womelsdorf’s lab	https://github.com/att-circ-contrl/wlBurst_v2
UMAP	Stephen Meehan	https://www.mathworks.com/fileexchange/71902
ISO-SPLIT	Chung et al. 2017^67^	https://github.com/flatironinstitute/isosplit5
SeNeCA	Tomek et al. 2013^68^	http://uemweb.biomed.cas.cz/tpp/
Spike sorting toolbox	Farries et al. 2023^69^	https://doi.org/10.5281/zenodo.8237447

### Experimental model and study participant details

#### Animals

All procedures involving animals were carried out in accordance with the guidelines of the Institutional Animal Care and Use Committee of the Department of Laboratory Animal Science at Fudan University (2021JS-NZHY-001). The mice were group-housed, with 4–5 animals per cage, in a temperature and humidity-controlled room under a 12:12 h light/dark cycle with lights on at 08:00. Mice of both sexes were used in all experiments, except for the *in vivo* recordings where only male mice were used. Mice were randomly assigned to sham and 6-OHDA groups. The number of mice used for each experiment were labeled in figures or figure legends.

The genetic background of all strains of mice used in this study (see key resources table) is C57BL/6J. PV-Cre mice (B6.129P2-Pvalb^tm1 (cre) Arbr^/J) and fluorescent reporter Ai9 mice (B6.CgGt (ROSA) 26Sor^tm9 (CAG tdTomato) Hze^/J) were purchased from The Jackson laboratory. These two genotypes of mice can be mated to obtain PV-Cre × Ai9 genotype mice, which were used to label PV-INs. D1-Cre mice (B6.FVB (Cg)-Tg(Drd1-Cre)EY262Gsat/Mmucd) and D2-Cre mice (B6.FVB(Cg)-Tg(Drd2-Cre)ER44Gsat/Mmucd) were purchased from Mutant Mouse Resource Research Centers (MMRRC). C57 adult mice were purchased from the Charles River Laboratories.

The mice in the sham and 6-OHDA groups were matched for age. For slice recordings, 6-OHDA injections or vehicle were given to animals when they were 4–8 weeks old. The subsequent experiments were initiated 1 to 8 weeks after the injection. Consequently, the majority of animals were around 5 to 16 weeks old on the day experimental data were collected. However, there was a subset of D2-cre mice (*N* = 5) that received 6-OHDA or vehicle injections at an age of 14–15 weeks, and these animals were approximately 20 weeks old at the time of the experiment.

For *in vivo* recordings, animals received 6-OHDA (or vehicle) injections and electrode implantations at an age of 6–10 weeks. The experiments were carried out 1 to 10 weeks after the injection, with the animals being approximately 7–20 weeks old at the time of the experiments.

### Method details

#### Stereotaxic injection of 6-OHDA and AAV viruses

Prior to surgery, desipramine (25 mg/kg i.p.) was administered 30 min before to minimize the uptake of toxin by noradrenergic and serotonergic axons. During surgery procedure, the mice were anesthetized with 1–2% isoflurane, with their body temperature continually monitored and kept around 37°C.

For lesioning, 6-OHDA (800 nL of 6-OHDA solution with a concentration of 4 mg/mL) or vehicle (saline solution containing 0.1% ascorbic acid) was injected into the MFB: 1.2 mm ML, 0.9 mm AP, 4.9 mm DV. To label target cell types for slice recording, we injected AAV viruses (300 nL, AAV2/9-Ef1a-DIO-EYFP-WPRE-hGH-pA) in the dorsolateral striatum: 2.2 mm ML, 0.20 mm AP, 2.42 mm DV. For pharmacogenetic experiments, we injected AAV viruses (200 nL, AAV2/9-hsyn-DIO-hM3D(Gq)-mCherry-WPRE-PA) into three sites in the striatum of both hemispheres, 1) 0.86 mm AP, ±1.77 mm ML, 2.42 mm DV, 2) 0.26 mm AP, ±2.11 mm ML, 2.42 mm DV, and 3) −0.34 mm AP, ±2.59 mm ML, 2.42 mm DV.

The 6-OHDA and vehicle solution were stored in the dark at −20°*C*, and the AAV viruses were frozen in the −80°*C* refrigerator. After surgery, mice were housed in home cage on a warming cabinet (27°*C*) and supplemented with high-fat pet milk daily as needed.

#### Striatal slices preparation

Mice were anesthetized using 1% sodium pentobarbital, and their brains were then transferred to ice-cold dissecting artificial cerebro-spinal fluid (ACSF). The dissecting ACSF consisted of 126 mM sucrose, 2.5 mM KCl, 2 mM MgSO_4_, 2 mM CaCl_2_, 26 mM NaHCO_3_, 1.25 mM NaH_2_PO_4_ and 25 mM dextrose (pH 7.4, 315 mOsm) and was saturated with 95% O_2_ and 5% CO_2_. Coronal slices (250 μm in thickness) were obtained using a Vibratome VT1200S (Leica, Wetzlar, Germany), and then transferred to recovery ACSF composed of 126 mM NaCl, 2.5 mM KCl, 2 mM MgSO_4_, 2 mM CaCl_2_, 26 mM NaHCO_3_, 1.25 mM NaH_2_PO_4_ and 25 mM dextrose (pH 7.4, 315 mOsm). The slices were incubated in recovery ACSF for 45 min at 34.5°*C*, continuously aerated with 95% O_2_ and 5% CO_2_, and then stored at room temperature until further use.

#### Whole-cell recording

The slices were transferred to a recording chamber, which was perfused with room temperature aerated ACSF (The ingredients are the same as recovery ACSF) at a rate of 1.2 mL/min. Neurons were visualized using an upright infrared differential interference contrast microscope (BX51WI, Olympus). The target cells (PV-INs, D1-SPNs and D2-SPNs) were identified by the expression of tdTomato, together with their firing pattern if possible. Recorded neurons were further subjected to post-hoc avidin staining.

The patch pipettes had an impedance of 5–7 MΩ. The intracellular solution in pipettes contained 138 mM Cs-CH_3_SO_3_, 3 mM CsCl, 2 mM MgCl_2_, 2 mM Na_2_ATP, 10mM HEPES, 0.2 mM EGTA, 4 mM QX-314, and 0.2% biocytin (pH 7.2, 286 mOsm). Whole-cell recording was achieved using a MultiClamp 700B amplifier (Molecular Devices, USA) and Micro1401 (Cambridge Electronic Design, UK) system. Signals were filtered at 10 kHz and sampled at either 25 or 50 kHz. EPSCs were recorded with holding potentials at −70 mV and IPSCs at 0 mV. To evoke postsynaptic currents, an ACSF-filled glass electrode was placed in the striatum approximately 100 μm away from the recorded neuron. Current pulses (10–30 μA in intensity, 0.1 ms in pulse duration, 20 Hz, eight pulses) were delivered using an isolator (Iso-Flex, A.M.P.I., current mode) controlled by a custom Spike2 script.

After forming a whole-cell recording, we waited for a period of time (about 5 min) to allow enough time for the blockers, QX-314 and cesium ion, to diffuse into the cell, as reflected by the absence of sodium spikes (i.e., action potentials mediated by the voltage-gated sodium channels). Most recordings were obtained within 20–30 min s after the membrane was ruptured to form whole-cell recording. The data length of sEPSC and sIPSC used for analysis spanned 100-2000s, matched between sham (359 ± 13 s, mean ± s.d.) and 6-OHDA groups (371 ± 13 s). Recordings with a holding current more negative than −200 pA when membrane potential were clamped at −70 mV, or a series resistance (Rs) of >25 MΩ were excluded from further analysis.

#### Immunofluorescence staining and image acquisition

In some experiments, we performed tyrosine hydroxylase (TH) and avidin staining on the same striatal slice that was previously subjected to electrophysiological recording. To do so, we stored the slices in 4% PFA-4% sucrose fixation solution for more than 24 h. At the first day of staining, we rinsed the slices with 0.01 M PBS three times (10–15 min each time), followed by incubation with 0.5% Triton solution for half an hour to partially permeabilize the membrane and expose the antigen epitope, and then blocked with a 5% bovine serum albumin solution containing 0.1% Triton for 1 h. After being rinsed, the slices were incubated overnight (usually 16 to 20 h) with a mixture of primary TH antibody and avidin. On the next day, the slices were rinsed and incubated with secondary antibody for 2 h. After another rinsing, we transferred the slices to a glass slide and dropped an anti-fade mounting medium. The above procedures were conducted at room temperature. The primary antibodies, secondary antibodies and avidin were diluted at a ratio of 1:1000. Bilateral striatum TH fluorescence images were acquired by an Olympus VS120 microscope using 10× objective. The z stack image (0.5 μm in step) of individual neuron was acquired by a confocal microscope (FV3000, Olympus) equipped with a 60× objective.

The experimental procedure for PV staining was similar to the above. The primary PV antibody was diluted at a ratio of 1:5000. The images were acquired by a confocal microscope (A1 plus, Nikon) equipped with a 10× air objective. Laser intensity and other imaging parameters were set during the acquisition of sham samples and kept identical for all images.

#### *In vivo* electrophysiology recording

To examine the alterations in spiking and LFP activity following dopamine depletion or pharmacogenetic activation of PV-INs, we inserted 4-tetrode bundle into the dorsolateral striatum (2.2 mm ML, 0.20 mm AP, 2.42 mm DV) during the same surgery in which 6-OHDA was injected into the ipsilateral MFB (1.2 mm ML, 0.9 mm AP, 4.9 mm DV). Each tetrode was composed of four polyimide-coated Ni-Chrome wires with an impedance of 250–500 kΩ (California fine wire). A resin head-plate was also affixed alongside the electrodes. For the CNO experiment, the pharmacogenetic viruses were injected into the striatum concurrently with electrodes implantation and 6-OHDA injection during a single surgical procedure. Three weeks after the surgery, we administered CNO to the animals and performed the recordings.

Recordings were conducted on head-fixed, awake mice. The animals were given 3–5 days to acclimate to a custom-made head-restraint device with 0.5–1 h of daily exposure prior to the experiment. Following acclimation, the mice exhibited minimal movement during recording sessions, and any data segments containing substantial motion artifacts were excluded from analysis. Recordings took place at a consistent time period each morning, with each animal being recorded for approximately 1 h continuously. Data were sampled with Apollo acquisition system (Bio-Signal Technologies, USA) and digitized at a rate of 30 kHz.

### Quantification and statistical analysis

#### Immunohistochemistry image analysis

Data were analyzed using MATLAB. For TH intensity analysis and cell localization, we first standardized the images by registering them into the Allen Mouse Brain Atlas using SHARCQ toolbox.^65^ On the standardized image, we drew an ROI in the right dorsolateral striatum (DLS) to obtain TH intensity on the lesion side. Since the images now become symmetrical after standardization, we flipped the ROI to the corresponding position on the left side of the DLS to obtain the TH intensity on the control side. The TH intensity ratio shown in Figure 1 was calculated by dividing the intensity on the lesion side by that of control side.

For PV intensity analysis, we utilized the SeNeCA algorithm^68^ to automatically extract each PV-IN soma and further manually edited if necessary. The SeNeCA algorithm consists of two steps: 1) identify the somatic centroids through local thresholding, and 2) expand the centroid to appropriate edge through flooding process. Parameters were set as follows: *highLightThreshold* = 1.25, *lowLightThreshold* = 2, and *maximumDiameter* = 80 μm. The resulting centroids were stored in an Excel file and manually edited when necessary. For somata not detected by the algorithm, step 1) was modified by forcing the centroid to be a manually selected point.

#### Spiking activity analysis

The detection and sorting of spikes were accomplished via a MATLAB-based implementation of MountainSort, developed by Berke’s laboratory.^69^ The results were refined with careful manual curation. To identify and analyze high-quality single units, we employed a set of stringent quality metrics: an amplitude greater than 50 μV, an inter-spike interval (ISI) violation of less than 0.5, a noise-cutoff of less than 0.5, and a signal-to-noise ratio (SNR) exceeding 2.5.

In an effort to differentiate between putative PV-INs and SPNs in an unsupervised fashion, we utilized UMAP for dimensionality reduction, coupled with the ISO-SPLIT clustering method.^67^ This approach classifies neurons into two main groups: narrow-spike and wide-spike neurons. Historically, in healthy animals, wide-spike neurons have been divided into ChINs and other subtypes,^70^^,^^71^ primarily distinguished by their initial positive phase^71^ or a tonic firing pattern.^70^ However, in parkinsonian animals, distinguishing ChINs is not straightforward. The initial positive phase is inconsistent across studies,^70^^,^^72^ and the firing patterns of ChINs evolve from tonic to irregular or burst-like with pauses in parkinsonian animals.^58^ Consequently, we did not further separate the wide-spike neurons. Notably, while ChINs exhibit an increase in bursting rate in parkinsonian condition, wide-spike neurons in our data show a decrease. Therefore, we believe that the impact of potential contamination from ChINs on the firing statistics of putative SPNs is minimal.

To quantify the bursting activity, we employed a ”burst index”, as previously described.^73^ The calculation of this index involved determining the sum of autocorrelogram counts within specific time frames—2-10 ms and 35–50 ms, referred to as the ”head” and ”tail”, respectively. The burst index was then derived by taking the difference between the head and tail values, normalized by dividing it by the sum of both.

#### LFP analysis

The LFP was extracted from raw data by down sampling to 1000 Hz and then bandpass filtering from 1 to 475 Hz. To compute power spectrum, the LFP was cut into non-overlapping 10-s segments. Any segment containing motion artifacts were removed. Power spectrum of all segments were computed and averaged using *mtspectrumc* function in Chronux toolbox^66^ with the parameters *TW* = 3 and *K* = 5. To account for potential variations in electrode impedance throughout the recording period, the average power spectrum was further normalized to its mean values between 1 and 90 Hz, avoiding 50 Hz line noise. Oscillatory burst events in LFP were detected using wlBurst_v2 toolbox (https://github.com/att-circ-contrl/wlBurst_v2). The toolbox was developed by Womelsdorf lab and is designed to detect and aggregative burst events from multi-electrode recording data. By using this toolbox, we set the parameters as follows: type = ”magdual”, qlong = 10, qdrop = 0.5, qglitch = 1.0, dbpeak = 6, and dbend = 2.

#### Spontaneous EPSC and IPSC analysis

Spontaneous synaptic current events were automatically detected and then manually checked. First of all, the baseline drift estimated by a moving median filter with 1 s window was subtracted from the raw trace to facilitate detection. Detection was achieved by using a hybrid of following methods. 1) Based on slope^74^: the slope of signal was calculated in a 1 ms window and then smoothed by a moving average filter with a 0.2 ms window. We then kept only negative slope points by accumulating them and setting positive slope points to zero to facilitate the detection of fast rising times of synaptic events. 2) Based on high-band amplitude^75^: the signal was first high-pass filtered above 10 Hz, then amplitude thresholding was applied where the threshold was set at 4.5 times the standard deviation. 3) Based on multi-band amplitude: unlike only high-frequency band in 2), we performed similar calculations for different bandpass filtered versions of the signal, with high cutoff frequency fixed at 300 Hz and low cutoff frequency varying 2–20 Hz. We did not adopt the slope-based method for IPSC detection, because this method cannot capture the slow rise time of IPSC. For traces with high signal-noise ratio, the above hybrid approach can detect almost all events and produce few false positive detections. But for noisy traces, the method is far from perfect and requires manual inspection.

#### Bootstrap resampling analysis of synaptic currents

To estimate relative change in parameters between sham and 6-OHDA groups, we performed a bootstrap resampling procedure. In each iteration, we randomly selected 75% of the total data from 6-OHDA group or sham group, calculated their average, and then divided (Figures 2G, S2C, S2D, S2F, and S2H) or subtracted (Figure 2H) the average of 6-OHDA group to that of sham. We repeat the above resampling process for 1000 times to calculate the 95% confidence interval and mean. If the mean does not fall within this interval, it is considered significant at the 95% confidence level.

#### Statistical analysis

Unless otherwise specified, the error bars in figures represent s.e.m. Welch’s t-test, two-way ANOVA, Kruskal-Wallis or Kolmogorov-Smirnov test was used for comparisons when appropriate. Datasets were considered to be significantly different if *p-value* was less than 0.05.
