# Supplementary material for: Early synaptic dysfunction of striatal parvalbumin interneurons in a mouse model of Parkinson’s disease
Source: iScience. 2024 Oct 24;27(11):111253. doi: 10.1016/j.isci.2024.111253 (PMC11575173; doi:10.1016/j.isci.2024.111253)
Supplement: Document S1. Figures S1–S5 [file mmc1.pdf]

iScience, Volume *27*

Supplemental information

Early synaptic dysfunction of striatal parvalbumin interneurons in a mouse model of Parkinson's disease

Quansheng He, Xiaowen Zhang, Hongyu Yang, Dahui Wang, Yousheng Shu, and Xuan Wang

Supplementary figure legends

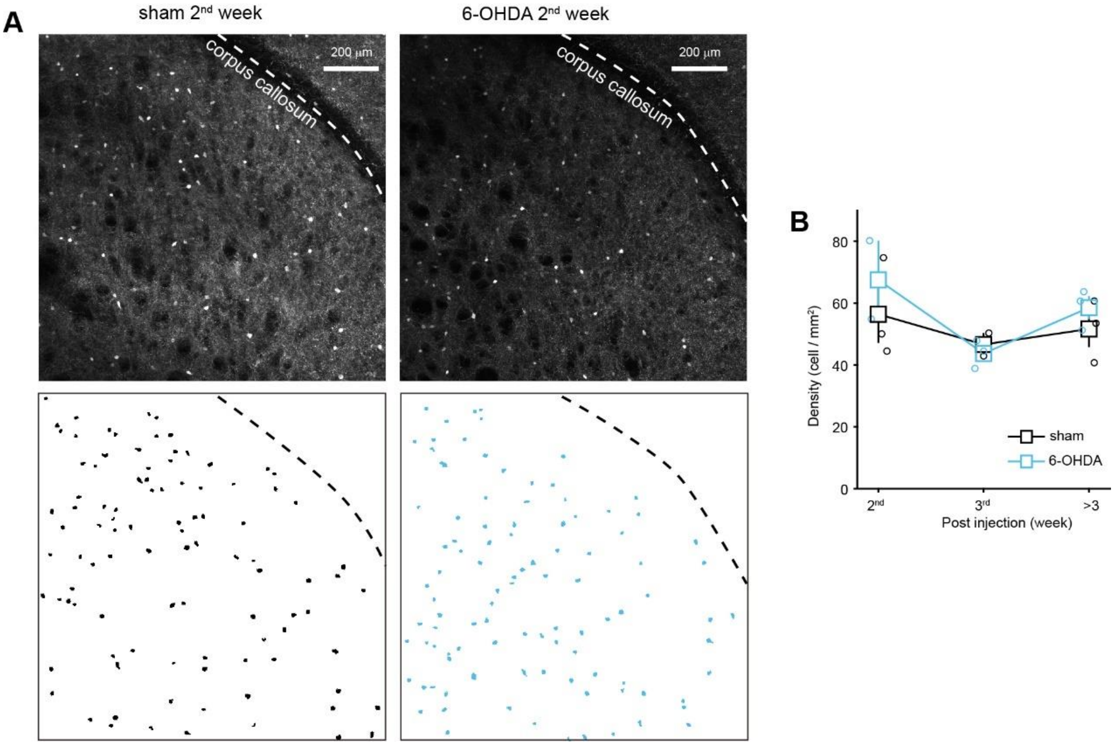

Figure S1. The density of striatal PV-INs remains unaltered in 6-OHDA-treated mice. Related to Figure 1

(A) Microscope images displaying PV staining are presented in the upper row, with the algorithmically identified locations of striatal PV-INs below.

(B) Group data showing the density of PV-INs across time windows.

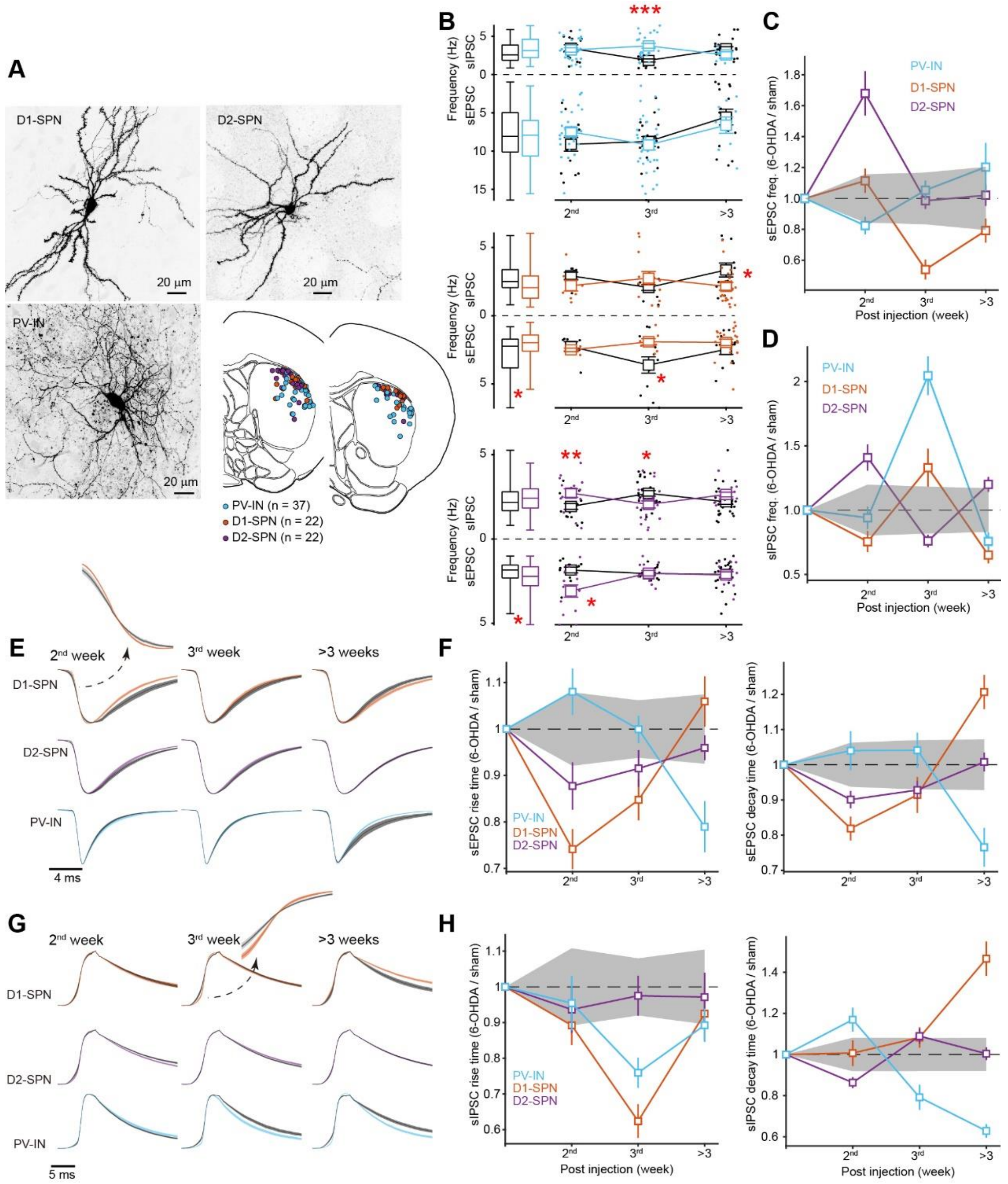

Figure S2. Locations of recorded neurons and spontaneous synaptic activity parameters beyond amplitude. Related to Figure 2

(A) Morphology and locations of whole-cell recorded striatal neurons.

(B) Pooled data across all time windows (box graphs) or pooled by specific time window (line graphs). (* $P < 0.05$, ** $P < 0.01$, *** $P < 0.001$, Welch's *t*-test).

(C-D) Relative changes in sEPSC and sIPSC frequency in three cell types.

Shading area indicates ± 3 SD from the mean of bootstrap samples.

(E) Scaled sEPSC events obtained from the three types of neurons in sham (black) and 6-OHDA (other colors) groups. Dotted arrows indicate the magnified rising phases.

(F) Relative changes in sEPSC rise (left) and decay time (right).

(G-H) Similar to panels (E-F), with data pertaining to sIPSC.

In panel C,D,F and H, the initial point on each curve, marked at a y-axis value of 1, is an artificial reference point introduced to aid in visualizing the effects of 6-OHDA.

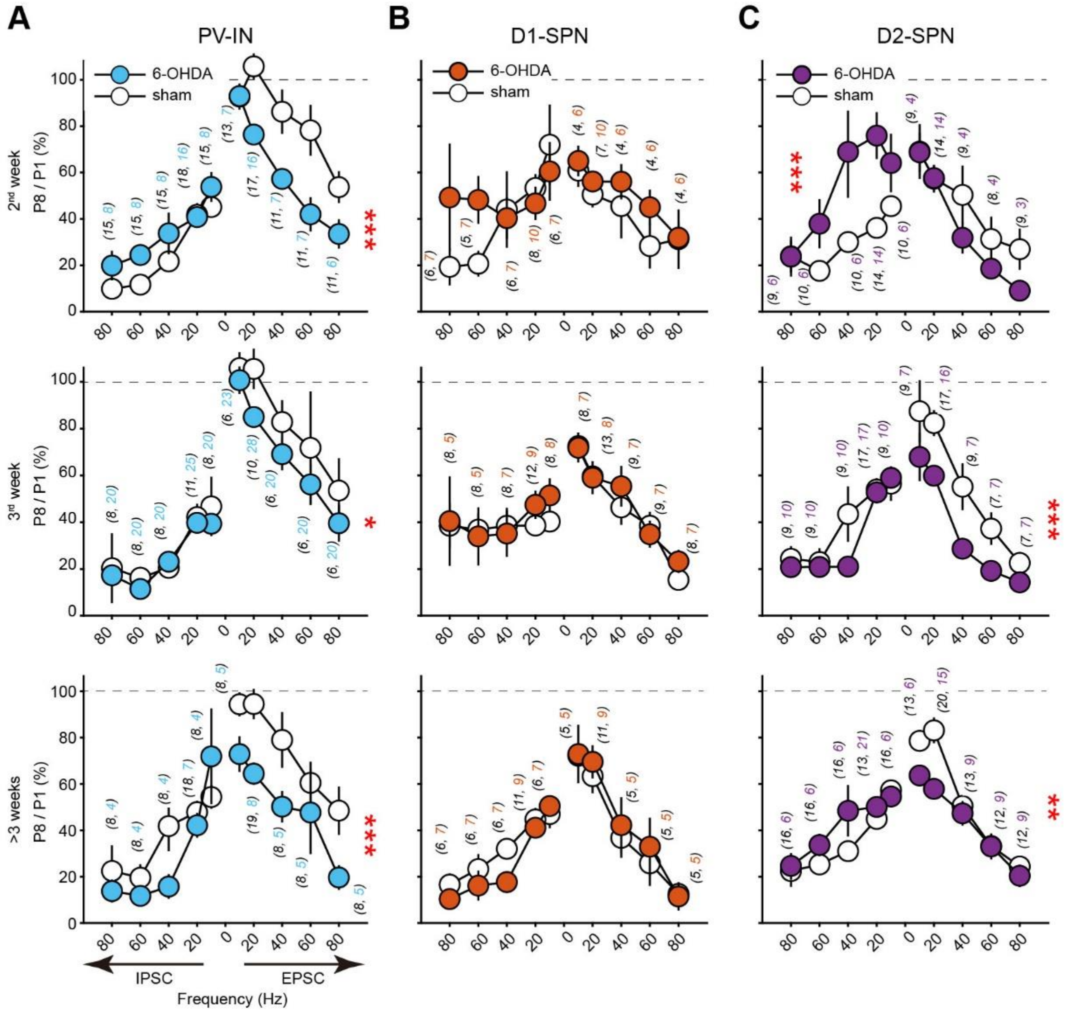

Figure S3. Short-term plasticity examined at various frequencies. **Related to Figure 3**

(A) The normalized 8th peak amplitude of EPSCs (rightward) and IPSCs (leftward) measured at various frequencies. (**P* < 0.05, ****P* < 0.001, two-way ANOVA. The number of cells is shown in a color-coded manner).

(B-C) Analogous data presentation for D1-SPNs and D2-SPNs, respectively. (***P* < 0.01, ****P* < 0.001, two-way ANOVA).

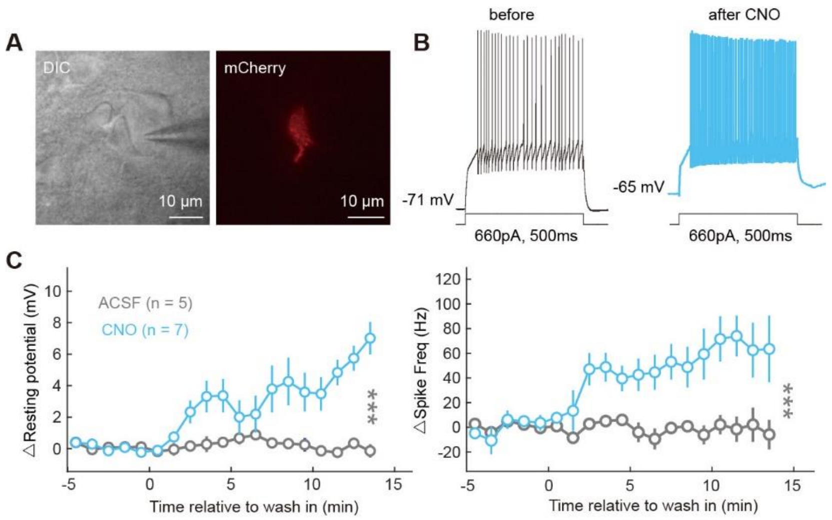

Figure S4. Validation of pharmacogenetic activation of PV-INs *in vitro.* Related to Figure 6

(A) DIC and mCherry images of a striatal PV-IN.

(B) Responses of a mCherry positive PV-IN to step current injections before and after the bath application of CNO (10 μM).

(C) Group data showing CNO-induced changes in the resting membrane potential (left) or the firing frequency evoked by current injections (right). The current intensity was adjusted for individual cell to allow firing at a frequency around 50 Hz before the application of CNO (****P* < 0.001, two-way ANOVA)

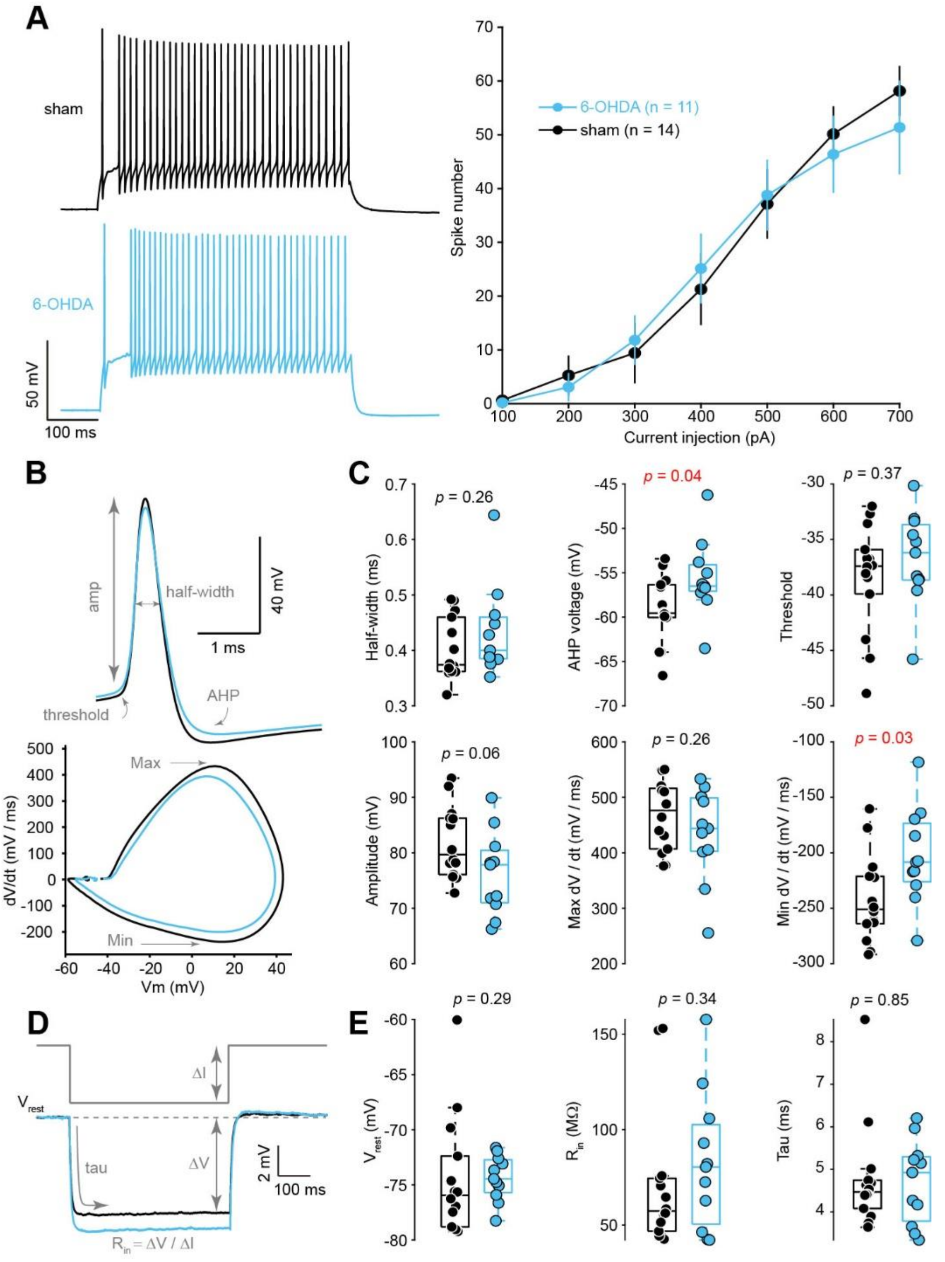

Figure S5. Subtle changes in cellular excitability of PV-INs.

(A) Left, representative action potential (AP) responses in PV-INs from sham and 6-OHDA-treated mice upon 500 ms, 500 pA current injection. Right, AP frequency vs. injected currents amplitude. (B) Average AP waveforms and their corresponding phase plots at the bottom. Six AP parameters used for comparison are illustrated.

(C) Comparative statistics for AP parameters in PV-INs from sham- and 6-

OHDA-treated mice (Welch's *t*-test). The *P*-values for two significantly altered parameters (AHP and dV/dt minimum) are highlighted in red.

(D) Average membrane potential responses to hyperpolarizing current injection from sham or 6-OHDA groups. Typical parameters used for comparison are illustrated.

(E) Membrane parameters of PV-INs from sham- and 6-OHDA-treated mice (Welch's *t*-test).
